# Translation directionality and the Inhibitory Control Model: a machine learning approach to an eye-tracking study

**DOI:** 10.3389/fpsyg.2023.1196910

**Published:** 2023-05-02

**Authors:** Vincent Chieh-Ying Chang, I-Fei Chen

**Affiliations:** ^1^Department of English, Tamkang University, New Taipei, Taiwan; ^2^Department of Management Sciences, Tamkang University, New Taipei, Taiwan

**Keywords:** cognitive load, pupillometry, machine learning, eye-tracking, translation asymmetry, directionality

## Abstract

**Introduction:**

Based on such physiological data as pupillometry collected in an eye-tracking experiment, the study has further confirmed the effect of directionality on cognitive loads during L1 and L2 textual translations by novice translators, a phenomenon called “translation asymmetry” suggested by the Inhibitory Control Model, while revealing that machine learning-based approaches can be usefully applied to the field of Cognitive Translation and Interpreting Studies.

**Methods:**

Directionality was the only factor guiding the eye-tracking experiment where 14 novice translators with the language combination of Chinese and English were recruited to conduct L1 and L2 translations while their pupillometry were recorded. They also filled out a Language and Translation Questionnaire with which categorical data on their demographics were obtained.

**Results:**

A nonparametric related-samples Wilcoxon signed rank test on pupillometry verified the effect of directionality, suggested by the model, during bilateral translations, verifying “translation asymmetry” at a *textual* level. Further, using the pupillometric data, together with the categorical information, the XGBoost machine-learning algorithm yielded a model that could reliably and effectively predict translation directions.

**Conclusion:**

The study has shown that translation asymmetry suggested by the model was valid at a *textual* level, and that machine learning-based approaches can be gainfully applied to Cognitive Translation and Interpreting Studies.

## 1. Research background

### 1.1. Positioning research perspective

#### 1.1.1. Process-oriented approaches to cognitive translation and interpreting studies

The study involves quantitatively measuring cognitive loading caused by directionality during *naturalistic* translation tasks, using such a method as eye-tracking. Therefore, this article is anchored in the emerging sub-area of “process-oriented” Cognitive Translation and Interpreting Studies (CTIS; see also Muñoz Martín and González Fernández, 2021; Chang, 2023) within the umbrella field of Translation and Interpreting Studies (for discussions on updated typological maps, see Van Doorslaer, 2007; Chesterman, 2009; Zanettin et al., 2015).

At the outset, it has to be stressed that the major goals of this paper are to support methodological innovation in CTIS and to provide a repeatable example of how to use multivariate approaches on pupil size data and multivariate data collected from a questionnaire described below. Given the emphasis on replication, the study relies on publicly accessible open-access text data and makes all findings available as a result. Reusable open data can, among other things, broaden perspectives (Fischer and Zigmond, 2010), foster collaboration (Zhu, 2020), and advance scientific progress and innovation (Borgman, 2012), all of which are essential to the developing field of CTIS. The selection of data for the analyses in the study is based on its emphasis on replication and openness.

Process-oriented CTIS has witnessed a number of recent key volumes presenting studies primarily based on “behavioral” evidence (e.g., Ferreira and Schwieter, 2014, 2015; Schwieter and Ferreira, 2017; Pease and Cheung, 2018; Li et al., 2019, 2022; Alves and Jakobsen, 2021; Carl, 2021; Wu et al., 2021; Zhang and Cheung, 2022; Chang, 2023; Liu X. et al., 2023). In contrast, the number of studies in process-oriented CTIS involving “physiological” data relatively pales in comparison. A limited number of such ground-breaking studies specifically evaluating cognitive loading have surfaced, despite the relative paucity of physiological research in CTIS. For instance, Li et al. (2023) measured the stress levels experienced by interpreters during crisis communication using a multimodal mix of physiological data including eye fixations, heart rates, and fluctuating levels of skin conductance. Additional investigations specifically exploring potential correlations between physiological measurements and cognitive loading during translation or interpreting tasks include those by Seeber (2013), Ehrensberger-Dow et al. (2020), and Gieshoff et al. (2021). Concurrently, while García (2019) and García et al. (2016) introduce neuroscientific research relating to translation and interpreting, Walker and Federici (2018) present eye-tracking studies in CTIS. Further, Shei and Gao (2018) specifically point out that, compared to behavioral studies, a relative paucity of research supported by physiologic al evidence warrants future CTIS research, particularly in the Chinese-speaking world (ibid.: 299).

Finally, it is worth noting that, in process-oriented CTIS, Rinne et al. (2000) have long recommended that further research using “less proficient students of translation studies as subjects” or novice translators as an under-researched population (ibid.: 88) would be useful (see also Rinne et al., 2007).

#### 1.1.2. Theory on “translation asymmetry”

The Inhibitory Control Model (ICM; Green, 1998) is a cognitive model of bilingualism, based on prior pioneering work by Kroll and Stewart (1994). The ICM posits that bilinguals have two language systems in their minds, which they must constantly inhibit or suppress to function in only one language at a time. The act of suppressing the stronger or more active system is cognitively demanding and results in a phenomenon called “translation asymmetry” (cf. Kroll and Stewart, 1994: 158), where L2 translation is more cognitively demanding than L1 translation. To clarify, for the use of terms relating to translation directions, the present study subscribes to Pavlović’s (2007) system of referring to “translating from one’s mother tongue into her/his second language” as “L2 translation” and “translating from one’s second language to her/his mother tongue” as “L1 translation.”

To further illustrate, the ICM (Green, 1998) argues that when we use a specific language, we activate the task schema for that language (Lx) and only allow lemmas (words) with the Lx language tag to be activated (cf. Schwieter et al., 2020: 84). This is done by increased activation of only those lemmas stored in the lexico-semantic system (Green, 1998: 67) that is equipped with the Lx language tag, while lemmas tagged for another language are inhibited. In addition to proactive activation of Lx, there is also reactive inhibition for contextually inappropriate lexical elements in Lx that might be activated erroneously. The task schema translates words from Lx to Language y (Ly), according to Green’s model, which performs three task schemata by specifying (1) Lx as the input language, (2) Ly as the output language, and (3) Lx as the language which must be inhibited to produce Ly. In other words, the ICM suggests that when we use one language, we actively inhibit any words from other languages that might interfere. Therefore, according to the ICM by Green (1998, see also Kroll et al., 2010: 8), during L2 translation, more effort is required to inhibit one’s mother tongue that has a stronger lemma system, while less effort needed to inhibit the weaker L2 system during L1 translation leads to the assumption that L2 translation is cognitively more demanding than L1 translation (see also Kroll et al., 2010).

The ICM has been supported by a large number of empirical “behavioral” studies, showing that bilinguals often perform better on cognitive tasks when they are asked to translate from their weaker language to their stronger language, compared to when they are translating in the opposite direction (e.g., Costa and Santesteban, 2004; Schwartz and Kroll, 2006; Dimitropoulou et al., 2011; Green and Abutalebi, 2013; Gray and Kiran, 2016; Darriba, 2019; Gonzalez Darriba, 2019). The explanatory term “translation asymmetry” has been used to refer to the inhibitory control demands of bilingualism, as it is assumed that bilinguals must constantly inhibit their stronger language during L2 translation. Such studies have confirmed that (translation) directionality, a central issue in Translation and Interpreting Studies (cf. Schwieter and Ferreira, 2017, p. 90–105; Baker and Saldanha, 2020, p. 152–156; Liu Y. et al., 2023), has an effect on cognitive loading, while L2 translation is generally considered more cognitively demanding than L1 translation (Pavlović, 2007; Chang et al., 2008; Chang, 2009; Chang, 2011), and that this could lead to lower translation quality (Whyatt, 2019). However, it is worth noting that “physiological” data to support “translation asymmetry” argued by the ICM are extremely limited. In fact, thus far, only one study (Korpal and Jankowiak, 2021) study has specifically used pupillometric data (measured in millimeters or mm) to test the effect of directionality on cognitive loading with moderate success, while two other studies (Christoffels, 2004; Christoffels and De Groot, 2009) have provided mere discussions regarding the potential usefulness of pupillometry as gainful measurements to test the same assumption.

Despite the success of the ICM in explaining many bilingual phenomena, it has been critiqued on a number of grounds. For example, some have argued that the “translation asymmetry” effect may be due to factors other than inhibitory control, e.g., differences in the difficulty of the two tasks (e.g., Brysbaert and Duyck, 2010). Moreover, the ICM does not explain why some bilinguals are better than others at inhibiting their stronger/weaker language (e.g., Bialystok et al., 2004). That being said, the ICM still remains an influential model of bilingualism and has provided a valuable theoretical framework with which to investigate varying cognitive loads accompanying “translation asymmetry” (cf. Kroll and Stewart, 1994: 158).

Further, to date, such studies on the effect of directionality on cognitive loading accompanying translation directionality have a number of weaknesses. First, although claims are made regarding the effect of improved proficiency, little work has been done with novice translators (see also Rinne et al., 2000, 2007). Second, the experimental tasks have involved only associations between *single words*, as opposed to *naturalistic* authentic translation tasks with *texts*. In fact, the ICM (Green, 1998) seems to assume that meaning resides in “single words” (lemmas); however, whether or not the predictions regarding “translation asymmetry” suggested by the ICM are valid at a *textual* level has yet to be explored. Third, a further criticism is that the distinction between lexical and conceptual links have been inferred from purely “behavioral” evidence (e.g., reaction times) and lacks direct “physiological” confirmation. This is why we report how we conducted an eye-tracking experiment to explore whether or not the predictions associated with translation asymmetry suggested by the ICM are valid at a “textual” level using physiological data collected from novice translators without a definitive hypothesis.

#### 1.1.3. Pupillometry and cognitive load

Eye-tracking data provides valuable insights into cognitive loads (e.g., Wang et al., 2014; Zu et al., 2020). Pupil size variations, pupillometric data or pupillometry are commonly used and well-established measurements of eye-tracking data, particularly helpful for assessing cognitive loads (cf. Ayres and Paas, 2012). Research has shown the usefulness of other eye-tracking data, e.g., the locations and number of fixations (Debue and Van De Leemput, 2014), fixation durations and saccadic lengths (Zagermann et al., 2016), eye blinks (Veltman and Gaillard, 1998) and blink latency (Zagermann et al., 2016).

Particularly, “pupillary response” is one of the most extensively researched measures of cognitive loads (see Klingner et al., 2008). Relationships between cognitive loading and pupil size have been found in many contexts, including simple cognitive tasks (Cohen et al., 2012), naval simulators (Greef et al., 2009), driving (Pfleging et al., 2016), e-learning (Porta et al., 2012), e-shopping (Jiang et al., 2021), and AI-assisted tasks (Chen et al., 2015). The pupillary reflex, unlike eye movement or blinking, is controlled by the autonomous nervous system (cf. Heller et al., 1990) and cannot be voluntarily controlled by the subject, which explains its relative objectiveness. However, this index suffers from its sensitivity to luminance variations (cf. Sirois and Brisson, 2014). Marshall (2002) has therefore suggested such a method as the use of a light meter to overcome the interference (ibid. for an in-depth explanation). In short, pupillometry has been long considered a reliable and objective measurement of cognitive loading in scientific endeavors in multiple fields (for detailed explanations, see Sirois and Brisson, 2014).

### 1.2. Machine learning-based approaches

Machine learning-based approaches to data analyses involve the adequate use and selection of algorithms that learn from datasets to make predictions or recommendations (cf. Lei et al., 2020). These algorithms can be used to detect patterns and trends in datasets, which can then be used to make predictions about current and future events or behaviors (cf. Mohammed et al., 2016). This type of approach has been successfully used in a variety of different disciplines, including medicine (Deo, 2015), finance (Dixon et al., 2020), and manufacturing (Wuest et al., 2016). In terms of task prediction based on cognitive load estimation, machine learning-based approaches have been shown to be more accurate than traditional statistical methods, such as chi-square tests or correlation analyses (cf. Alpaydin, 2020). Furthermore, machine learning-based approaches can be used in real-time *naturalistic* settings (e.g., Stone and Veloso, 2000; Rehtanz, 2003).

Machine learning, a subfield of artificial intelligence, deals with the design and development of algorithms that can *learn* from datasets and improve their performance over time (Ongsulee, 2017). A growing interest in using machine learning-based approaches has emerged to predict task types correlated with cognitive loading (Ayres and Paas, 2012). However, in CTIS, very limited research has been done to apply machine-based learning approaches to analyze empirical data associated with translation and interpreting tasks, with only very few exceptions (e.g., Baroni and Bernardini, 2006, related to translation, Michael et al., 2020, related to the use of machine translation, Ustaszewski, 2021, related to indirect translation), alongside conceptual discussions on how machine translation and machine learning can inform each other (see Schaeffer et al., 2020; O’brien, 2022). Still, whether or not machine learning-based approaches have the potential to be adequately used in the translation and interpreting field to more accurately predict the task type correlating with cognitive loading has yet to be explored and verified.

Further, machine learning-based approaches can be used to identify patterns in datasets, numerical as well as categorical, that are associated with cognitive loading (Abdul et al., 2020). For instance, if a machine learning-based algorithm is trained on a dataset of pupillometric data in combination with language/translation background information of research participants, it may potentially be able to learn which factors are associated with high cognitive load (e.g., relating to L2 translation) and which factors are associated with low cognitive load (e.g., relating to L1 translation). It is worth stressing that Balling (2008: 190) has explicitly demonstrated that newly developed statistical techniques can be effectively used to analyze such physiological data as pupil size variations accompanying *naturalistic* experimental translation tasks; therefore, Balling’s work (ibid.) serves as a foundation on which the present study explores if such new analytical techniques as machine learning-based analyses, in comparison to traditional statistical techniques, can be gainfully applied to predict translation directions with a relatively higher level of accuracy (see also Carl et al., 2016).

### 1.3. Research questions

The study therefore aims to answer two research questions (RQ):

RQ 1: Can the predictions suggested by the Inhibitory Control Model associated with translation asymmetry be valid at a *textual* level for naturalist L1 and L2 translations performed by novice translators?

RQ 2: Can machine learning-based approaches be adequately used to reliably predict the effect of translation directions using pupillometric data collected in the naturalist L1 and L2 translations?

## 2. Methodology

To answer the four research questions, the study conducted a within-subject repeated measures eye-tracking experiment, drawing on prior works by Chang (2009, 2011). Because “directionality” was the only factor guiding the experimental design, the experiment involved a total of two tasks, i.e., L1 and L2 translations, while such within-participant repeated-measures dependent variables as pupillometry were measured accordingly during the bilateral translations.

### 2.1. Eye-tracking experiment

#### 2.1.1. Participants

With an IRB approval, 14 bilingual participants who had finished the first year of their postgraduate translation training in universities in the UK, with Mandarin Chinese as their mother tongue and English as their second language participated in the experiment with informed consent. They were all female, with three of them from Taiwan and the other 11 from Chinese Mainland. The age bracket of nine of them fell between 18 and 25, while that of the other five, 26 and 35. The model of the eye-tracker is ASL 504 Model Monocular Eye-tracker (50 hertz). Before the experiment started, all the participate were requested to sign a consent form (Appendix 1) and fill in a Language and Translation Questionnaire (Appendix 2). Questions regarding the participants’ language and translation background that are normally considered factors impacting translators’ performances were included to obtain further *qualitative* details of the participants, and the information described variability between the individuals involved (see Brown, 1988; Bradburn et al., 2004; Chang, 2005). Another potential value that might result from the Questionnaire was that categorical data, e.g., gender, as well as numerical data, e.g., the age at which a participant started learning English as a second language, could be factored in for machine learning-based data-driven analyses later in the study. All the participants were paid an honorarium for their participation in the 90-min-long experiment.

Each experimental session involved a repeated measures experimental design with a total of two tasks: (1) L1 translation, using a short English text (Appendix 3); and (2) L2 translation, using a short Chinese text (Appendix 4). In the repeated measures experiment, during L1 and L2 translations, pupil size data variations over time in each participant were recorded. Because both tasks were, respectively, completed under 5 min, each participant yielded at least 12,000 data points.

In addition to this, a screen-recording software was used to ensure that the participants carried out the tasks as instructed. The task sequence across the participants was randomized. Further, before a participant carried out a particular task, the researcher presented the texts to the participant to read out aloud *once*. This was done because a translator is generally recommended to read a text to be translated in advance before s/he starts to translate it.

For the reasons listed below, reading aloud was chosen as a warm-up activity before the translation tasks. Although translators in naturalistic settings would not normally read aloud a text prior to a translation task, reading aloud prior to a translation task is an effective method for ensuring that the translator focuses on processing the source text at hand. By reading aloud, participants are given time to internalize the content before they begin their translations, which allows them to better comprehend and prepare for the task ahead. Additionally, by having participants read out loud, experimenters can more easily identify any potential issues with comprehension, allowing them to intervene and provide necessary support. This is especially important for learners whose first language may be different from the source text being translated. Hence, while silent reading is certainly an option for a translator to prepare for the task at hand, reading aloud provides an additional opportunity to engage with the source text in a more immersive and comprehensive manner. Finally, through reading out loud, participants are encouraged to attend more closely and carefully to the language used in both sides of the translation as well as being conscious of the words they will need to use in their translation. Thus, reading aloud was selected as it provides a better understanding of the participant’s comprehension than if they were to read silently.

#### 2.1.2. Material design and selection

Text selection was a critical aspect of the study design. Several elements were considered, in terms of criteria for text selection. First, the study aims to achieve as much *ecological validity* as possible (see Seeber et al., 2019; Baekelandt and Defrancq, 2021). “Ecological validity” refers to how well an experimental task reflects the *real-world naturalistic* situation under study and how well the experimental results can be generalized to the real-life scenario under investigation (cf. Gile, 2013; Ehrensberger-Dow et al., 2015; Mellinger and Hanson, 2022). Therefore, to *maximize* ecological validity of the study, we used controlled written “texts,” as opposed to “single words,” as stimuli to *simulate* real-life naturalistic situations.

While it is important to strive for as much ecological validity as possible in such a study, there are certain practical limitations that make it difficult to achieve perfect ecological validity. For instance, the experimental setup does not entirely replicate naturalistic translation settings in terms of the environment, participant’s language proficiency, and task familiarity. Additionally, due to the limited duration of the experiment, only a small subset of texts was chosen for the experiment, making it difficult to generalize results across different text types or contexts. However, despite these limitations, we believe that our design effectively captures the relevant cognitive processes involved in translation directionality and is able to provide useful insights into the effects of this phenomenon on cognitive load. Furthermore, our findings are consistent with previous research that has examined the dynamics of eye movements during reading and translation tasks (Chang, 2008; Chang, 2009; Chang, 2011). In conclusion, while the study does not achieve perfect ecological validity, it still makes a meaningful contribution towards our understanding of the effects of translation directionality on cognitive load.

On a different note, the present study is interested only in predicting “translation directions” using pupillometric data that correlate with cognitive loading, simple and non-technical texts will be selected, while exploring if predictions regarding “translation asymmetry,” suggested by Green (1998), are valid at a *textual* level. It is therefore necessary to exclude possible text-related confounding variables; therefore, simple and non-technical texts were tested. The Mandarin language was chosen from a Mandarin textbook (Lai, 2006) used in Taiwan, and the English text was taken from a single children’s storybook (Milne, 1980).

Moreover, we conducted “word count,” “grade level,” “readability,” “comprehensibility,” and “translatability” tests to ensure that (1) the potential effect of any of the above factors was *teased out* or at the very least *minimized*; and (2) the two texts were comparable, with “directionality” as the only independent variable in the experimental design (cf. Chang, 2011, p. 162–4).

##### 2.1.2.1. Word count

An orthographic principle word as it is used in English is distinct from the concept of a word in a language with ideograms, like Mandarin, where words are a compound unit that is equivalent to a letter that is frequently made up of two English word unit. As an illustration, the Chinese character “火” indicates “fire” and the character “車” means “vehicle.” Combining the two characters would result in a compound word “火車,” meaning “train.”

The word count test was conducted to ensure that the two texts in question had an equal number of words, eliminating the potential effect of differing word counts. The English text was modified using Microsoft Word to get it down to exactly 50 words, while two Mandarin language and translation teachers independently calculated the word count of the selected Mandarin text. After careful consideration and modifications without affecting the grammar or meaning, they reached a consensus that the Mandarin text also consisted of 50 “words.” In this way, the potential effect of differing word counts was minimized.

##### 2.1.2.2. Grade level

The grade level test was conducted to ensure that the English and Mandarin texts were both usable and understandable by pupils in the same educational stage. The Flesch–Kincaid Grade Level Test, which could calculate a score for English text, resulted in a reading level of 4.5, corresponding to the second semester of grade four in an elementary school in the US educational system. As this test could not calculate scores for Mandarin texts, a Mandarin textbook used in the second semester of grade 4 in Taiwan was obtained instead to match the English text’s grade level. The short text extracted from the textbook then enabled a comparison between the two texts with regards to their grade level and minimized any text-related difficulty factors.

##### 2.1.2.3. Readability, comprehensibility, and translatability

In order to determine readability, comprehensibility and translatability levels of the texts, two professional translation teachers were recruited. Both had Mandarin as their first language and English as their second. The teachers read both texts while rating them on a scale from 1 (very easy) to 5 (very difficult). After thorough iterative deliberation, they gave both texts a score of 1 on each test with an inter-rater reliability (k) of 1 reached on the readability, comprehensibility and translatability tests, confirming that both texts were simple to read, comprehend and translate.

## 3. Empirical results

Results from the screen-recording software as well as behavioral data have shown that all the participants conducted the tasks as instructed, whereby confirming the validity of the pupillometric data for further analyses (cf. Chang, 2011, p. 168–9).

### 3.1. Related-samples Wilcoxon signed rank test on pupillometry

First, to identify problematic pupil size values, each participant’s time series data was examined. After visual inspection of the eye-tracking recordings, it was possible to identify abrupt changes in the pupil size during recording due to blinks and fixations on other elements besides the screen stimuli. Through this analysis, any potential slips or errors committed by participants were identified and excluded from the data analysis. Additionally, any zeros that occurred due to an over-blinking of the eyes was also flagged and excluded. As a result of this process, at least 12,000 valid data points were obtained for each participant in the study - even after excluding all problematic pupil size values.

Next, due to the relatively small number of participants recruited and a lack of normal distribution associated with the pupillometric data, a nonparametric related-samples Wilcoxon signed rank test has revealed that the overall pupil size of L2 translation (mean = 4.46, STD = 1.18, median = 4.53) was significantly larger than L1 translation (mean = 4.36, STD = 0.60, median = 4.19, W statistics = 87.00, *p* = 0.03). This has revealed that, at a *textual* level, L2 translation was more cognitively demanding than L1 translation, and that predictions associated with translation asymmetry suggested by the ICM were valid at a *textual* level. This has answered RQ 1.

### 3.2. Traditional statistical analyses using pupillometry and questionnaire data

To firstly explore the usefulness of traditional statistical technique (cf. Stevens, 2012) for analysis in the present study, F-tests, Chi-square tests and a correlation analysis tests were conducted (see also Bartholomew et al., 2008). As shown in Table 1, a total of 16 predicting variables from the Questionnaire were included for analyses in combination with the repeated-measures pupillometric data. In the case where categorical data was concerned, conversion into numerical notations was completed. The 16 variables were: (1) pupil size at the very beginning of a task or START PUPIL; (2) pupil size at the very end of a task, the average of pupil size or END PUPIL; (3) standard deviation of pupil size variations or PUPIL STD; (4) the average pupil size throughout a task or AVERAGE PUPIL; (5) the age at which a participant started acquiring English as a foreign language or STARTING AGE; (6) the total number of months spent studying English or LEARNING MONTHS; (7) the total number months spent using English in a practical way or PRACTICING MONTHS; (8) the total number months being immersed in an English-speaking environment or IMMERSION MONTHS; (9) English test scores or SCORES; (10) the proportion of L1 translation done during the training in the current program or EC; (11) the proportion of L2 translation done during the training in the current program or CE; (12) Nationality; (13) age or AGE RANGE; (14) Handedness, starting age of learning English; (15) the translation direction a participant feels more comfortable with or COMFORTABLE; and (16) the total number months ever spent working as a translator on a paid professional basis or PRO BASIS. Factoring in all the 16 variables in combination with the repeated-measures pupillometric data with each participant.

**Table 1:** Descriptive statistics of the 16 predicting variables.

	Mean	Std. Deviation	Mode	Percentiles		
				25	50	75
Numerical variables
1. Start pupil	5.109	1.008	4.63	4.63	5.25	5.72
2. End pupil	4.892	0.776	4.38	4.38	4.815	5.38
3. Pupil STD	1.561	0.732	0.818	1.168	1.377	1.879
4. Average pupil	4.407	0.9182	1.222	3.953	4.441	5.037
5. Starting age	10.214	2.853	12.0	9.0	11.5	12.0
6. Learning months	130.143	39.775	108.0	108.0	132.0	156.0
7. Practicing months	90.5	39.280	36.0	60.0	89.0	132.0
8. Immersion months	28.214	25.728	9.0	9.0	16.0	46.0
9. Scores	6.964	0.592	6.5	6.5	7.0	7.5
10. EC	0.880	0.206	0.5	0.5	0.9	1.0
11. CE	0.196	0.206	0.0	0.0	0.1	0.5
Categorical variables
12. Nationality	-	-	2	2	2	2
13. Age range	-	-	1	1	1	2
14. Handedness	-	-	1	1	1	1
15. Comfortable	-	-	1	1	1	3
16. Pro basis	-	-	1	1	1	2

We firstly performed F-test, Chi-square tests and a contingency coefficient point-biserial Pearson *r* correlation analysis explore whether or not the paired relationships of each of the 16 predicting variable and the independent variable (directionality) were significant (*p* < 0.05). As shown in Table 2, no such significant associations were revealed. In other words, all of the paired relationships were statistically independent. Because this suggests that traditional statistical techniques could not identify potential interrelationships among the multiple factors in the current dataset, we subsequently performed a machine leaning-based classification tree algorithm as an analytic tool with which to train the dataset to see if potential results regarding predicting translation directions could be yielded.

**Table 2:** Correlation analysis.

Paired variables (numerical*categorical)	F-test (*p-*value)	Point-Biserial Correlation (*p-*value)
Start pupil * GROUP	0.255 (0.618)	0.98 (0.618)
End pupil * GROUP	1.001 (0.326)	−0.193 (0.326)
Pupil STD * GROUP	0.121 (0.731)	0.068 (0.731)
Average pupil * GROUP	0.086 (0.772)	0.057 (0.772)
Starting age * GROUP	0.00 (1.00)	0.00 (1.00)
Learning months * GROUP	0.00 (1.00)	0.00 (1.00)
Practicing months * GROUP	0.00 (1.00)	0.00 (1.00)
Immersion months * GROUP	0.00 (1.00)	0.00 (1.00)
Scores * GROUP	0.00 (1.00)	0.00 (1.00)
EC * GROUP	0.00 (1.00)	0.00 (1.00)
CE * GROUP	0.00 (1.00)	0.00 (1.00)
Paired variables (numerical*categorical)	Chi-square test (*p*-value)	Contingency Coefficient (*p*-value)
Nationality * GROUP	0.00 (1.00)	0.00 (1.00)
Age range * GROUP	0.00 (1.00)	0.00 (1.00)
Handedness * GROUP	0.00 (1.00)	0.00 (1.00)
Comfortable * GROUP	0.00 (1.00)	0.00 (1.00)
Pro basis * GROUP	0.00 (1.00)	0.00 (1.00)

GROUP is the target variable indicating translation directionality, with group 1 = L1 translation and group 2 = L2 translation.

### 3.3. Machine learning-based analyses: Prediction model using pupillometry and questionnaire data

The above results using the traditional statistical analyses yielded no significant results, possibly due two issues that had yet to be addressed. First, the inter-individual variability associated with language/translation background may impact the predictive power of a model across all the participants (Yin and Zhang, 2017). Second, it has to be acknowledged that potential participant-dependent artifacts in real-time pupillometric data collection may reduce the signal-to-noise ratio, whereby reducing *accuracy* of the cross-subject predictive power of the model. Therefore, we decided use machine learning to construct a demographic-pupillometry model for predicting translation directions, using both the pupillometric alongside the 16 factors.

Additionally, unlike traditional statistical methods, machine learning is able to construct and train a prediction model that may explain non-linear relationships and extract features from complex, high-dimensional data (Chen and Lu, 2021) without strong *a priori* assumptions. Moreover, while the “decision tree approach” is among the most widely used machine learning techniques (cf. Alpaydin, 2020), one of the most important decision tree algorithms is “random tree” (RT: Quinlan, 1986). By utilizing information-theoretic measurements like entropy, RT can assess potentially significant associations hidden in multidimensional datasets (Bresfelean, 2007). Further, the XGBoost algorithm is an improved variant of the traditional RT-based algorithms. Due to is its powerful ability to conduct large-scale parallel-boosted tree calculations using the instance-based induction learning method (for detailed technical explanations, see, e.g., Lantz, 2019; Alpaydin, 2020), we selected XGBoost from the constellation of machine learning analytic techniques for model implementation.

### 3.4. Model implementation

In this study, a task prediction model was created using XGBoosting as the classifier. The first step of the suggested modeling technique was gathering the data, including pupillometry data from an eye-tracker utilized in an eye-tracking experiment and demographic profiling data from a questionnaire. As indicated in Section 3.2. there were in total 16 predicting variables. The XGBossting tree-based prediction model was simple to understand and comprehensive thanks to the display of its outcomes, unlike many other machine learning methods. The Python XGboost module 1.7.0 and the sklearn package were used to create this XGboost-based prediction model, which was then displayed using the Python graphviz and matpotlib libraries.

The cutting-edge XGBoosting classifier was able to extract significant features of these two translation directions by using the instance-based induction learning method to match the sample data. In the current study, the XGBoosting model was built using a leave-one-out validation strategy to prevent the over-fitting issue and guarantee a desired generalization of the classifier. The model was iteratively trained on all other examples during this learning process and tested on the chosen instance, until each instance once served as the tested sample.

Using the leave-one-out validation method (cf. Zhou, 2021), a total of 28 testing iterations resulted in a model with an accuracy rate of 92.86% for predicting translation directions, while the recall rate was 85.71% with the F1-score of 0.9230. Table 3 shows that 100% of the 14 L2 instances were correctly predicted as L2 translation, while 12 of the 14 L1 instances were accurately predicted as L1 translation.

**Table 3:** Classification confusion matrix.

Class	Predicted L1 translation	Predicted L2 translation
Actual L1 translation	12	2
Actual L2 translation	0	14

**Figure 1:**
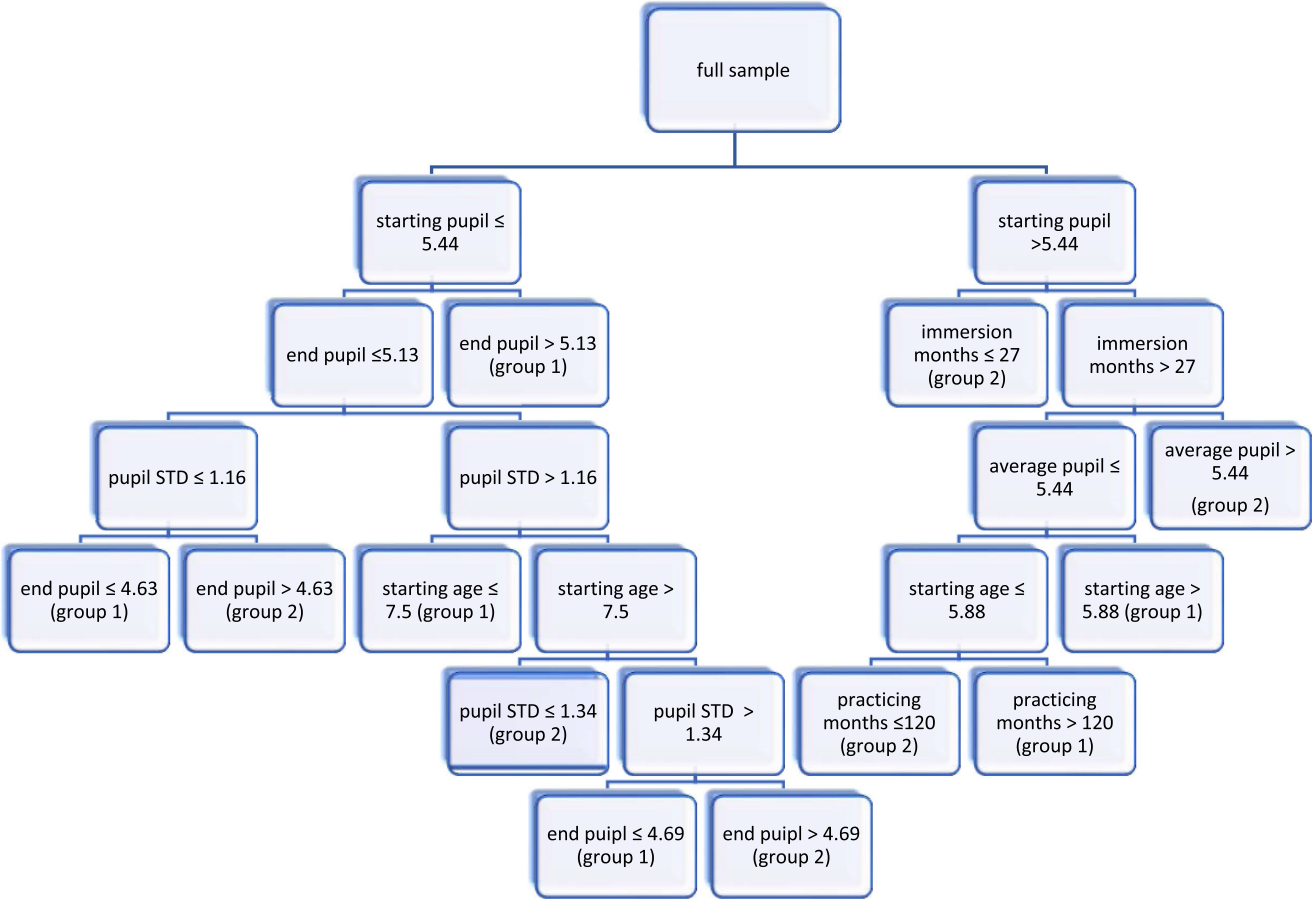
Visualization of the prediction model. group 1 = L1 translation and group 2 = L2 translation.

In the form of a tree-based prediction model to visualize the results, Figure 1 illustrates, with the machine learning model, the classification rules on which the instance-based predictions regarding L1 or L2 translation were made.

Using XGBoosting from the constellation of machine learning-based approaches to analyze the dataset in the present study, our results have furnished a model with an accuracy rate of 92.86% for adequately and reliably predicting translation directions. This has answered RQ 2.

The values of the model implementation are manifested by the present study, which demonstrates that a demographic-pupillometry prediction model using machine learning approaches can accurately and reliably predict translation directionality. This supports previous findings on the Inhibitory Control Model (ICM) in terms of language directionality. Furthermore, this improved predictive power demonstrated by the model can inform Computational Translation and Information Science (CTIS) in terms of development of better machine translation systems that take into account individual language proficiency, cognitive load, and other factors. Therefore, the present study highlights the importance of machine learning approaches in predicting translation directionality.

To further elucidate, the model implementation yielded highly encouraging results, with an accuracy rate of 92.86%, a recall rate of 85.71%, and an F1-score of 0.9230. This suggests that the Inhibitory Control Model applied to pupillometry data combined with questionnaire demographic information is effective at predicting translation directionality in CTIS learners. Furthermore, the results of the model are highly encouraging, as 100% of L2 instances were accurately predicted and 12 of 14 L1 instances were correctly classified as such. These findings suggest that pupillometry combined with demographic information is a useful tool for assessing CTIS learners’ language processing skills in terms of directionality.

## 4. Discussion

Via the study, we have discovered that seven out of the 16 predicting variables were important factors, and they are: (1) START PUPIL; (2) END PUPIL; (3) PUPIL STD; (4) AVERAGE PUPIL; (5) IMMERSION MONTHS; (6) STARTING AGE; and (7) PRACTICING MONTHS. This to a certain extent suggests that the seven factors may helpfully inform the prediction of translation directions, particularly when using machine learning for analyzing naturalistic translation tasks (for how numerical and categorical factors affect machine learning analytics, see Jordan and Mitchell, 2015).

Based on the values used *recursively* in Figure 1, further discussions are presented as follows. First, the machine learning XGBoosting technique has shown that L1 translation could be predicted when START PUPIL was smaller than 5.44, and END PUPIL was above 5.13. Regardless, this suggests a dwindling cognitive load during L1 translation (e.g., Pavlović, 2010). Second, when END PUPIL was below 5.13 with PUPIL STD below 1.16, then the value of END PUPIL would more likely fall under 4.63, suggest s/he was conducting L1 translation. However, when PUPIL STD fluctuated between 1.16 and 1.34 with the STARTING AGE over 7.5, the value of END PUPIL remained smaller than 4.69 for L1 translation. This suggests L2 translation was more cognitively demanding than L1 translation and lends further support to “translation asymmetry” argued by Green (1998) and Kroll and Stewart (1994).

On the other hand, when START PUPIL was larger than 5.44 with less than 27 IMMERSION MONTHS, the machine learning predicted that the participant was conducting L2 translation. This further suggests that, in this case, L2 translation was more cognitively demanding than L1 translation (cf. Green and Abutalebi, 2013). Additionally, when IMMERSION MONTHS surpassed 27 and PRACTICING MONTHS lasted longer than 120, the machine learning model predicted that the participant was conducting L2 translation, with her/his START PUPIL above 5.88 as well as AVERAGE PUPIL under 5.44. This suggests that their work experience as a professional translator to a certain extent had an effect on cognitive loading (see, e.g., Hunziker Heeb, 2020); in other words, those with more work professional experience found L2 translation less cognitively demanding, compared to the other participants with fewer months working as professional translators (see Neubert, 2000; Whyatt, 2018).

Via the present study, machine learning has been shown to be gainfully useful in the field of Cognitive Translation and Interpretation Studies (CTIS). This is demonstrated by our study, where a machine learning XGBoosting technique was used to accurately predict translation directions with an accuracy rate of 92.86%. Our analysis showed that seven out of the 16 predicting variables (including START PUPIL, END PUPIL, PUPIL STD, AVERAGE PUPIL, IMMERSION MONTHS, STARTING AGE, and PRACTICING MONTHS) were important factors in the prediction of translation direction. Furthermore, our results revealed that L2 translation was more cognitively demanding than L1 translation, and that work experience as a professional translator had an effect on cognitive loading. Making use of machine learning is therefore beneficial in the field of CTIS, allowing for more accurate predictions to be made based on various variables.

Our study has employed a machine learning approach to analyze naturalistic translation tasks. This allowed us to accurately predict translation directions with an accuracy rate of 92.86%, as well as identify the important factors of START PUPIL, END PUPIL, PUPIL STD, AVERAGE PUPIL, IMMERSION MONTHS, STARTING AGE and PRACTICING MONTHS. These results provide insights into the cognitive load associated with translation directionality and how professional experience affects it. Through this interdisciplinary approach, machine learning has opened up new avenues for the further exploration of the relationship between directionality and cognitive load in translation tasks. Additionally, our results provide evidence that several factors such as pupil size, immersion months, starting age, and practicing months have a significant effect on cognitive loading in translation tasks. This demonstrates machine learning’s potential to gainfully provide insights into the cognitive processes of CTIS.

Future work could focus on optimizing the predictive power of machine learning-based approaches to CTIS. This might involve additional data collection, such as using a higher-hertz eye-tracker and incorporating control groups of professional translators. Additionally, other methods could be employed to analyze pupillometric data, such as time series analysis or event-related potentials. Additionally, the use of machine learning for directional control should be further explored in other translation and interpreting settings. Ultimately, future research should strive to more deeply understand the implications of directionality in order to gain insights into how this factor can be optimized through training and development programs. To summarize, these proposed areas of future work could potentially lead to deeper insights into the effect of directionality on cognitive load and translation efficiency. Such insights could be useful for practitioners, trainers, and researchers in the fields of CTIS. Future research could expand upon the findings of this study by exploring the influence of other variables on cognitive load and directionality in translation. Additionally, it would be beneficial to explore how pupillometry can be used alongside other physiological measures to provide a more comprehensive picture of the cognitive processes underlying translation. Finally, further studies should investigate how these findings can be applied in real-world contexts to improve translation process and quality.

## 5. Limitations and conclusions

### 5.1. Limitations

The limitations of the present study are as follows. First, due to limited availability, a relatively low-hertz eye-tracker was used. Despite this, reasonably abundant pupillometric data was sufficiently obtained for analyses in the study presented. However, a higher-hertz eye-tracker may be helpful in informing future research. Second, aside from pupillometric data, the study concurrently relied on self-reported questionnaire data, which may potentially have been subject to subjectivity bias impacting the results. Finally, the study did not include a control group. Had a control group of professional translators been added, the predictive power of the machine learning model might have been diminished, due to a lesser degree of difference accompanying cognitive loads. Despite these limitations, the present study has provided valuable insights into the potential gainful application of machine learning-based approaches to CTIS, while advancing our understanding of how ICM can be applied and integrated into CTIS.

### 5.2. Conclusion

The significant and novel contributions to knowledge are manifold. First, methodologically, in CTIS, the study is one of the first few studies that may demonstrate potentially gainful application of machine learning techniques to pupillometric data to predict translation directions. Second, ontologically, the experiment involves recruiting participants from the relatively under-researched novice group. Third, epistemologically, the study may potentially inform the theoretical integration of ICM and CTIS. Fourth, application-wise, if it can be determined that L2 translation is cognitively more demanding than L1 translation, this suggests that incorporating more L2 translation training to help students cope with market challenges (Hao and Pym, 2021) is required as a way of optimizing the training. Finally, as for societal impact, with the potentially optimized training, this matches the UN Sustainable Development Goal 4 of Quality Education (cf. Boeren, 2019).

The present study has provided physiological evidence for the conclusion that translation asymmetry or the effect of directionality, suggested by the Inhibitory Control Model (Green, 1998), is valid at a textual level. In addition, the study’s findings suggest that machine learning-based approaches can be gainfully applied to the field of CTIS. This is because the machine learning modeling as shown in the study successfully predicted translation directions, using pupillometry together with data from the Questionnaire. Consequently, these findings provide support for the conclusion that machine learning-based approaches can be used to validly and reliably estimate differing cognitive loads accompanying L1 and L2 translations. Furthermore, the findings of the present study suggest that machine learning-based approaches can be used to predict cognitive load associated with directionality with a high degree of accuracy. Consequently, these findings have implications for the potential use of machine learning-based approaches to estimate cognitive loading in other translation and interpreting settings.

Further to this, the present study has contributed to a deeper understanding of the ICM at a text level by demonstrating the physiological differences between L1 and L2 translation tasks. Through the use of pupillometry, we were able to obtain direct physiological evidence that translation asymmetry is valid in translation tasks, thus supporting ICM’s notion of directionality on a textual level. Concurrently, the study has also provided evidence of the theoretical as well as methodological values of incorporating physiological measurements in translation studies. This is because, while self-reported questionnaires can provide information on participants’ subjective perception of cognitive loading, physiological measurements such as pupillometry are able to provide direct and objective evidence regarding how the cognitive processes behind translation activities affect the body. In addition, by combining pupillometric data with self-reported questionnaire data in this study, we were able to gain a better understanding of how participant-related factors such as age and language proficiency influence cognitive loading during translation tasks. Finally, the findings of the present study suggest that machine learning-based approaches can be used to accurately predict cognitive loading associated with directionality, with the potential of being applied and integrated into CTIS in a more effective way. Consequently, these findings have implications for the use of machine learning-based approaches not only in translation and interpreting settings, but also in other fields where cognitive load estimation is necessary. To summarize, the present study has yielded substantial evidence that directionality has a direct physiological impact on cognitive load, and that machine learning-based approaches can be used to accurately estimate this effect with great accuracy.

In other words, this study has provided valuable insights into the role of translation directionality in translation processes as well as established new potentials for machine learning-based approaches to be applied in this field. These findings contribute to a deeper understanding of the inhibitory control model at textual level and have important implications concerning the use of physiological measurements and participant-related factors in translation studies.

To conclude, our research has contributed to the field of CTIS by advancing our understanding of how ICM can be applied and integrated into CTIS in a more effective way, as well as providing insights into how physiological measurements can be used to better understand the cognitive processes behind translation activities. As a result, this research has provided essential and invaluable information for both practitioners and researchers alike.

## Data availability statement

The datasets presented in this study can be found in online repositories. The names of the repository/repositories and accession number(s) can be found at: https://doi.org/10.7910/DVN/Y1MKH9.

## Ethics statement

The studies involving human participants were reviewed and approved by Imperial College London. The patients/participants provided their written informed consent to participate in this study.

## Author contributions

This study is an extension and continuation of the Chang’s doctoral research, the research presented in the manuscript is a separate study, on the epistemological, ontological and methodological levels. Therefore, VC essentially designed the whole study, ran the entire eye-tracking experiment, collected the questionnaire data, and wrote up the manuscript. I-FC completed the machine learning analysis as well as the nonparametric related-samples Wilcoxon signed rank test on pupillometry while furnishing a drafted discussion on the analyses. The discussion was then fine-tuned by VC. All authors contributed to the article and approved the submitted version.

## Conflict of interest

The authors declare that the research was conducted in the absence of any commercial or financial relationships that could be construed as a potential conflict of interest.

## Publisher’s note

All claims expressed in this article are solely those of the authors and do not necessarily represent those of their affiliated organizations, or those of the publisher, the editors and the reviewers. Any product that may be evaluated in this article, or claim that may be made by its manufacturer, is not guaranteed or endorsed by the publisher.
