# Supplementary material for: Translation directionality and the Inhibitory Control Model: a machine learning approach to an eye-tracking study
Source: Front Psychol. 2023 May 2;14:1196910. doi: 10.3389/fpsyg.2023.1196910 (PMC10187886; doi:10.3389/fpsyg.2023.1196910)
Supplement: Supplementary file 1 [file Data_Sheet_1.docx]

**Appendix 1. Consent form**

**Consent Form for Translators: An Eye-tracking Study**

You are invited to participate in a study of translations. My name is Vincent Chieh-Ying Chang, an assistant professor at the Department of English, Tamkang University, New Taipei City, Taiwan. In this study, I hope to investigate how translation directionality relates to cognitive effort during translating. You have been selected as a potential participant because you are a translation student with the language pair of Chinese and English. You are one of the students in translation- or interpretation-related programs in universities or colleges who have been invited to participate in this study.

If you decide to participate, you will be asked to fill out a questionnaire on your demographic information, your IELTS score, age of second language acquisition, handedness, your translation training, prior educational background and work experience. You will be asked to conduct translation of two 50-word non-technical texts, one into English and the other into Chinese, alongside two typing tasks respectively in English and Chinese for the sake of practice. You will be asked to sit in front of a computer, with a non-invasive infrared eye-tracker recording your eye movement and pupil dilation. The whole process will take xxx minutes at most. Before starting the eye-tracking tasks, you will be requested to sign a consent form. Should you have any questions, please do not hesitate to ask the investigator. When you have finished the translations, you will be given 1,000 NTD as honorarium for your participation. Any personal information obtained in this study will remain confidential.

I want you to understand that your participation in this study is completely voluntary and that you are free to choose not to participate. If you do choose to participate, you may withdraw at any time during the study simply by telling me that you no longer wish to participate. Your decision whether or not to participate will not affect your future relations with the Department of English or Tamkang University.

If, having read the above information, you decide to participate, please sign below to indicate that you have understood all of the above and that you give your consent to participate in this study as it is described.

Please feel free to ask me any questions before or after signing this consent form. If you have any other questions later, you may contact me at +886(0)2 2621 5656 ext.: 3236, Department of English, No. 151, Yingzhuan Rd., Tamsui District, New Taipei City, Taiwan. I will be happy to answer all your questions. You will be offered a copy of this form.
