# Supplementary material for: Translation directionality and the Inhibitory Control Model: a machine learning approach to an eye-tracking study
Source: Front Psychol. 2023 May 2;14:1196910. doi: 10.3389/fpsyg.2023.1196910 (PMC10187886; doi:10.3389/fpsyg.2023.1196910)
Supplement: Supplementary file 2 [file Data_Sheet_2.docx]

**Appendix 2. Language and Translation Background Questionnaire**

**Language and Translation Background Questionnaire**

*This questionnaire concerns your language and translation experiences over the course of your lifetime. You can choose to answer the following questions either in Chinese or in English. All responses are confidential and will be analyzed only for research purposes. If there are questions you prefer not to answer, you may choose to skip them. Thank you for your participation.*

**PARTICIPANT __________**

1. Where were you born? __________

2. What is your nationality? __________

3. What is your gender? Male / Female

4. What is your age?

18-25 26-35 36-45 46-55 56-65 over 65

5. What is your handedness? Right-handed /Left-handed

6. What is your native language? __________

7. What is your second language? __________

8. What is your language of habitual use now? __________

9. What form of Chinese are you more comfortable with during reading?

Simplified Chinese / Traditional Chinese

10. What form of Chinese are you more comfortable with during writing?

Simplified Chinese / Traditional Chinese

11. What other languages or dialects do you know (including both foreign languages and dialects such as Cantonese, Taiwanese or Hakka, etc.)? _________________________________________________________

12. What is the Chinese input method that you are used to using on a computer? _______________

13. Which language do you find more difficult to key in on a computer and why?

A. English / B. Chinese

Please explain: ______________________________________

14. At what age did you start learning your second language? __________

15. Up to today, what is the total amount of time you have spent on formally studying your second language in a school setting? ______ year(s)__________month(s)

16. Up to today, what is the total amount of time you have spent using your second language in a practical way (e.g. verbal communication, paper writing, reading for obtainment of knowledge, etc.)? __________year(s)__________month(s)

17. Up to today, what is the total amount of time you have spent being immersed in an English-speaking environment? ___________year(s)__________month(s)

18. In what major was your **undergraduate** degree? __________

19. What is your overall band on IELTS? _______

20. What are the bands for different tests in IELTS? (Or test scores on CET, TOEIC or TOEFL)

A. Listening _____ B. Reading _____

C. Writing _____ D. Speaking _____

21. Which university or college do or did you attend for your postgraduate translation training? ____________________

22. How long is the **postgraduate** translation course mentioned in Question 21? ­­­__________

23. Of all the **postgraduate** translation assignments, approximately how much is translating from English into Chinese and how much from Chinese into English?

From English into Chinese _____%

From Chinese into English _____%

24. Which DIRECTION of translation do you find more comfortable working with?

A. From English into Chinese

B. From Chinese into English

25. Have you ever worked as a translator on a professional basis (e.g. paid translation work)?

Yes (Please proceed to answer the next question).

No (Please proceed to answer Question 28.)

26. How long have you worked as a professional translator? __________

27. Of all the professional translation assignments, approximately how much is translating from English into Chinese and how much from Chinese into English?

From English into Chinese _____%

From Chinese into English _____%

28. What (other) work experiences have you had so far? ___________________________________________

THANK YOU FOR FILLING IN THIS QUESTIONNAIRE!

Participant’s Signature __________ Researcher’s Signature __________ Date:_________
