# Supplementary material for: Translation directionality and the Inhibitory Control Model: a machine learning approach to an eye-tracking study
Source: Front Psychol. 2023 May 2;14:1196910. doi: 10.3389/fpsyg.2023.1196910 (PMC10187886; doi:10.3389/fpsyg.2023.1196910)
Supplement: Supplementary file 3 [file Data_Sheet_3.docx]

**Appendix 3. English text for L1 translation**

**Final exams are held in June at universities and colleges across Taiwan. Today, on the grass in front of the exam room, a student is asleep. His two companions are seated by him. These three are first-year university students. They will take their final exams later in the same classroom.**
