# Supplementary material for: Translation directionality and the Inhibitory Control Model: a machine learning approach to an eye-tracking study
Source: Front Psychol. 2023 May 2;14:1196910. doi: 10.3389/fpsyg.2023.1196910 (PMC10187886; doi:10.3389/fpsyg.2023.1196910)
Supplement: Supplementary file 4 [file Data_Sheet_4.docx]

**Appendix 4. Chinese text for L2 translation**

**有一乞丐，對他自己現在的生活，抱怨連連；於是，他決定去拜訪開心的人，請教他們讓自己開心的方法。他聽了他們許多的故事與建議，發現這些人開心的方法說到底，其實都是一樣的。那就是：知行合一，想到自己喜歡的事情，就立刻採取行動去追求。**
